# Looking into New Sources of Bioactives: Seasonal Variation in Bioactive Compounds and Dietary Fiber of Agave Bagasse from Mezcal Production

**DOI:** 10.3390/foods14091632

**Published:** 2025-05-06

**Authors:** Jimena Álvarez-Chávez, Elisa Dufoo-Hurtado, Liliana Santos-Zea, Aurea K. Ramírez-Jiménez

**Affiliations:** School of Engineering and Science, Tecnologico de Monterrey, Av. Eugenio Garza Sada 2501 Sur, Monterrey C.P. 64849, NL, Mexico; jimena.alvarez@tec.mx (J.Á.-C.); elisa.dufoo@tec.mx (E.D.-H.); lilianasantos@tec.mx (L.S.-Z.)

**Keywords:** *Agave* spp., bagasse, seasons, phytochemical, bioactive compounds, FTIR, waste, by-products

## Abstract

The production of mezcal from agave is one of the main beverage industries worldwide, generating large amounts of waste, such as agave bagasse. Improper management of this waste often causes environmental contamination. Some studies have begun to demonstrate that agave bagasse has the potential to be recycled as a source of functional ingredients due to its dietary fiber and bioactive compound content. However, the greatest disadvantage of using these wastes is the significant variation in compound content and bioactivity in response to seasonal climatic variations. This study aimed to analyze the chemical and bioactive content of agave bagasse from three mezcal factories in Mexico. We conducted proximate composition analysis, phenolic compound measurement, dietary fiber assessment, antioxidant capacity evaluation, and structural analysis using FTIR- Fourier Transform Infrared Spectroscopy. The study found significantly higher ash content (17.75%), carbohydrates (86.71%), and soluble fiber (30.91%) in the spring compared to other seasons. The summer showed a higher lipid content (10.25%), while the highest concentration of sugars (47.77%) was observed during the winter. The highest antioxidant capacity (106.15 mM eq Trolox/mg) was recorded in autumn. The FTIR analysis revealed that the greatest abundance of bioactive compounds was observed in the spring and summer, while structural carbohydrates were more prominent in autumn and winter. This study is the first to thoroughly analyze the seasonal variation in phytochemicals and macromolecules in agave residues, crucial for exploring new ingredient sources to expand our food supply and recycling agri-food wastes.

## 1. Introduction

Humanity is at a key point in solving environmental and sustainable food problems. The agricultural industry produces 182 billion tons of lignocellulosic biomass annually worldwide, of which only 4.4% is reused [1]. Lignocellulosic waste contains lignin (10–20%), hemicellulose (15–35%), and cellulose (30–50%) [2], which could be harnessed in a circular economy process, for example, in the food industry, as a source of bioactive compounds and functional ingredients.

In Latin America, the agave plant holds great importance as it produces various beverages, both distilled and non-distilled, including mezcal, tequila, pulque, sotol, and bacanora. Mexico stands out as one of the leading producers of alcoholic beverages derived from agave, exporting tequila and mezcal worth USD 4.429 billion in 2023 [3]. From a more worldwide perspective, agave is a source of natural sweeteners, providing an alternative to table sugar and a vegan alternative to honey [4]. However, the amount of waste generated from agave production poses significant environmental and economic challenges that require proper management.

Mezcal, the second most consumed alcoholic beverage after tequila, is crafted using an artisanal process, depending on its place of origin. Mezcal production involves waiting 7 to 10 years for the agave to mature. The plant’s leaves, locally known as “pencas”, are cut to concentrate the sugars in the plant’s core (leaf base). The core is then removed from the ground and cooked in an earthen oven with wood and agave leaves for several days. Following this, the cooked core is manually macerated until it dissolves, followed by a 6-to-10-day fermentation process with drinking water and agave yeasts. The fermentation duration varies depending on the climate. Finally, the liquid undergoes distillation in wooden alembics; this is sometimes performed twice (double distillation) to increase the alcohol concentration. A similar process, without the fermentation and distillation, is carried out to hydrolyze the oligosaccharides for the production of agave sweetener [4].

Global mezcal consumption has significantly increased recently, with 66% of mezcal exported abroad in 2020. The United States is the largest importer, receiving 73% of mezcal, followed by Spain, Canada, and Australia. Moreover, mezcal has gained popularity in China, with exports reported to 72 countries [5]. This rise in demand stems from consumers’ preferences for artisanal crafted beverages.

More than 8 million liters of mezcal are produced annually in Mexico, with the State of Mexico, particularly the Malinalco region, accounting for 70% of its production [6]. The large-scale production of mezcal generates different types of waste, including bagasse, the fibrous residue that remains after fermentation and distillation. Unfortunately, mezcal producers often resort to improper disposal practices, such as burning or leaving it to decompose in open fields or rivers. When bagasse is burned, particulate matter and greenhouse gases are generated. Regarding decomposition in open fields or rivers, water pollution and growth of harmful microorganisms may result.

Agave bagasse is a lignocellulosic material and may still contain bioactive molecules. In this regard, there are no studies on the use of mezcal bagasse to obtain ingredients for human consumption, with only one study on aguamiel bagasse in this context [7]. As for the chemical composition of mezcal bagasse, only one study has detailed the levels of structural carbohydrates, sugars, and ashes of cooked agave bagasse (following the NREL- National Renewable Energy Laboratory procedures). It is noteworthy that the cooked agave bagasse underwent sugar extraction before analysis, which could have affected the measured carbohydrate content [8]. Previous studies have revealed that tequila bagasse consists of glucan (35%), xylan (15%), lignin (13%), cellulose (40%), and protein (3.5%) [9]. However, there is a lack of reports on the specific compoition of mezcal bagasse. To date, there is no information on the fiber and sugar contents, phenolic compound profiles, or antioxidant capacity of this material, nor on the variations of these compounds through the seasons of the year. Therefore, conducting a chemical characterization of the mezcal agave bagasse represents an advance in knowledge for this agro-industrial waste.

There is extensive information on the influence of the environment on the concentration of bioactive compounds and nutrients in plants. Nevertheless, there is scarce information on lignocellulosic residues, particularly on agave, despite its potential as a source of bioactive compounds. Seasonal changes, including climatic variations, solar radiation, and growing conditions, influence bioactive compounds and nutrients [10]. Prinsloo and Nogemane [11] reported that seasonal variation and water availability significantly changed the expression of genes related to primary and secondary metabolite production in various plants. For instance, summer conditions induce higher sugar and proline production in certain plant species, whereas secondary metabolites, such as terpenes, flavonoids, and phenolic acids vary from summer to autumn. Furthermore, during rainy seasons plants produce higher amounts of alkaloids, steroids, and carbohydrates. Concerning agave, only one study has reported differences in secondary metabolites, focusing on phenolic compounds and saponins for different agave ripening stages in the whole plant [12]. The results showed that mature plants had lower concentrations of metabolites, particularly saponins, which may affect the bioactivity they exert. Understanding these fluctuations allows us to identify the periods when these compounds are most scarce in lignocellulosic materials and when they have their maximum levels. Identifying the seasons when plants contain higher concentrations of bioactive compounds is crucial for optimizing collection and standardizing processing practices for waste.

This research aims to analyze the changes in the bioactive compounds of agave bagasse from mezcal production throughout the four seasons of the year (spring, summer, autumn, and winter). Agave bagasse was sourced from mezcal producers in the Malinalco region, State of Mexico—specifically from “La Cascada”, “El Palmar”, and “La Escondida”—selected for their significant influence on the region’s economy and environmental landscape. These studies are invaluable for the food industry in developing products with enhanced stability and quality year round. The proximate composition is determined, with quantification of sugars, dietary fiber, and phenolic compounds and antioxidant compounds; a vibrational analysis FTIR is also performed.

## 2. Materials and Methods

### 2.1. Sample Collection

**Figure 1:**
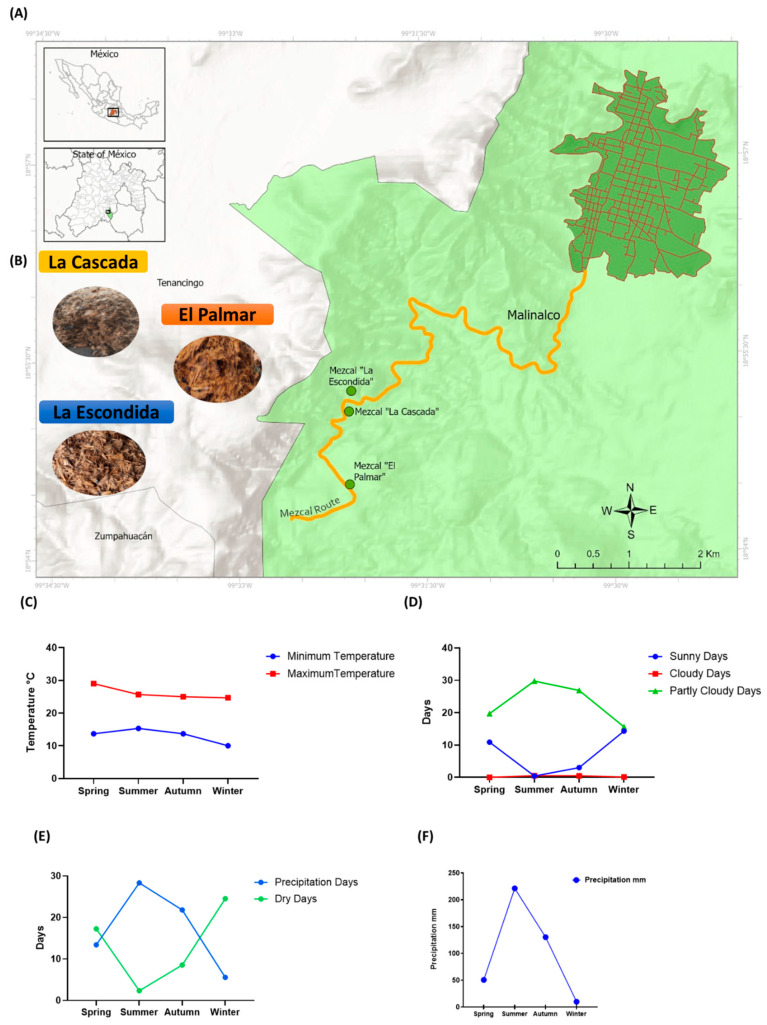
(**A**) Sites of the 3 mezcal factories located in Malinalco, State of Mexico, Mexico. (**B**) Agave bagasse of three different mezcal factories after the production of mezcal. (**C**) Maximum and minimum temperatures of Malinalco in spring, summer, autumn, and winter in 2023. (**D**) Sunny, cloudy, and partly cloudy days in Malinalco during the four seasons of the year in 2023. (**E**) Precipitation and dry days in Malinalco during the seasons of the year in 2023. (**F**) Precipitation in mm in Malinalco during the four seasons of the year in 2023. Information obtained from MeteoBlue software.

Three mezcal factories—”La Cascada”, “El Palmar”, and “La Escondida”—in the region of Malinalco in the State of Mexico provided bagasse collected throughout the four seasons of the year (spring, summer, autumn, and winter) in 2023. Each study involved three replicates for each sample, from three different mezcal producers and the 4 seasons of the year, resulting in 12 samples. Figure 1 shows the location of agave bagasse from the 3 mezcal factories; the environmental conditions of Malinalco in the four seasons of the year in 2023; the minimum and maximum temperatures during the seasons; the sunny, cloudy, and partially cloudy days; the precipitation and dry days during the four seasons; and the amount of rainfall during the four seasons. The samples were collected after the mezcal production. The agave bagasse was stored in sealed plastic bags and maintained at −70 °C to preserve its integrity until further analysis.

### 2.2. Sample Preparation

The agave bagasse, initially containing 90% moisture, was dehydrated using an dehydrator ( Model 2400, Excalibur, CA, USA) at 50 °C for 12 h until it reached a moisture level of 6%. Then, the sample was milled with a CNA-923C grain polisher (CGoldenwall, Los Angeles, CA, USA) until a powder with a particle size of 400 µm was obtained. The resulting material was stored in airtight plastic bags at room temperature.

### 2.3. Chemical Composition

The chemical composition was determined using the AOAC methods [13]. The moisture content was determined using method 325.23; the lipid content was quantified using method 920.39; and the ash content was measured by method 942.05. Method 46.16.01 was used to determine the protein content, and the total carbohydrates were calculated by subtracting the sum from the overall total. The results are reported as dry weight percentage (%).

### 2.4. Determination of Total and Reducing Sugars

For both experiments, the sample was diluted in water at a 1:10 ratio from a previously prepared liquid extract. To determine the total sugars we used DuBois’ phenol–sulfuric acid method [14] and compared the results to the standard glucose curve; it was measured at a wavelength of 490 nm with a spectrophotometer (Perkin Elmer Lambda 25, Shelton, CT, USA). To quantify the reducing sugars, we used Miller’s DNS (3,5-dinitrosalicylic acid) method [15] and the results were compared to a standard dextrose curve at 550 nm. In both cases, the results are expressed as dry weight percentage (%).

### 2.5. Analysis of Dietary Fiber Content

The AOAC [16] guidelines were followed for measuring soluble dietary fiber (method 991.43) and insoluble dietary fiber (method 985.29), using the Total Kit Megazyme Dietary Fiber Assay “K-TDFR-200A” (Megazyme International, Bray, Wicklow, Ireland). A phosphate buffer with pH 6 was used. First, 50 µL of α-amylase was added and incubated for 30 min at 98–100 °C. It was cooled to 60 °C and the pH was measured to be 7.5; then, 100 µL of protease was added and incubated for 30 min at 60 °C. It was brought to room temperature and the pH was measured to be 4. Finally, 200 µL of amyloglucosidase was added and incubated for 30 min. For insoluble fiber, it was vacuum-filtered with Whatman No. 4 paper, then washed with water (70 °C), ethanol (78%), and acetone. For soluble fiber, ethanol (95%, 60 °C) was added, then it was vacuum-filtered with Whatman No. 42 paper, and washes were performed with ethanol (95%) and acetone. The results are indicated as dry weight percentage (% dw).

### 2.6. Extraction of Free Phenolic Compounds

To obtain the free phenolic compounds, 10 mg of dry sample was extracted using 1 mL of methanol. The sample was shaken and sonicated for 30 min at 40 kHz using an ultrasonic bath model 5510 (Branson, Danbury, CT, USA). It was then centrifuged for 5 min at room temperature at 1300× *g* using a MiniSpin Plus centrifuge (Eppendorf, Hamburg, Germany), and the supernatant was subsequently collected and stored at −70 °C for later use.

### 2.7. Extraction of Bound Phenolic Compounds

The bound phenolics remaining after the extraction of the free phenolics were obtained from the pellet using 0.5 mL of 2 M NaOH. To avoid degradation by oxygen, the mixture was subjected to 30 min of sonication followed by 5 min of vortexing, following the method proposed by Zavala-López and García-Lara [17], with slight modifications. Finally, samples were preserved at −70 °C until further analysis.

### 2.8. Analysis of Total, Free, and Bound Phenolic Acid Contents

The total phenols were determined in the free and bound fractions using the Folin–Ciocalteu reagent, as described by Ainsworth and Gillespie [18]. To 100 µL of sample, 200 µL of Folin–Ciocalteu reagent and 800 µL of sodium carbonate were added; it was left to stand in the dark for 2 h and read at 765 nm with a spectrophotometer (Perkin Elmer Lambda 25, Shelton, CT, USA). A gallic acid standard curve was employed to compare the results, which were expressed as mg GAE/mL.

### 2.9. Antioxidant Capacity Analysis

The antioxidant capacity was evaluated in the methanolic extract using 2,2-diphenyl-1-picrylhydrazyl (DPPH) [19] and 2,2′-azinobis-3-ethyl-benzo-thiazoline-6-sulfonic acid (ABTS) radicals [20]. A standard Trolox curve was used to interpret the results, which were expressed as mM eq. Trolox/mg.

### 2.10. Vibrational Analysis

The agave bagasse samples were oven-dried to remove residual moisture, then ground and sieved to obtain a uniform particle size of approximately 400 µm. The prepared powder was analyzed using a Perkin Elmer IR spectrometer (Spectrum Two, Waltham, MA, USA) equipped with an attenuated total reflectance (ATR) accessory. Infrared spectra were collected in the range of 600 to 4000 cm^−1^, with 32 scans accumulated per sample.

### 2.11. Statistical Analysis

All samples were analyzed in triplicate, and the results were expressed as mean ± standard deviation (SD). Statistical analyses included one-way and two-way ANOVA, applying a significance threshold of 0.05. Tukey’s test was used for post hoc comparisons to detect significant differences among means. Prior to these tests, all datasets were assessed for normality and homogeneity. The analyses were conducted using the Minitab Statistical Software (Minitab 22.2.1, LLC, 2021, State College, PA, USA). Additionally, principal component analysis (PCA) was carried out with the vegan package in R 4.2.3 (R Package, LLC, 2024, Oulu, Finland).

## 3. Results and Discussion

### 3.1. Chemical Composition

Seasonal changes in the bagasse of *Agave angustifolia* from mezcal have yet to be studied. Table 1 shows the chemical composition of agave bagasse collected from three mezcal factories in Mexico across different seasons (spring, summer, autumn, and winter). The moisture content shows significant variation depending on the season and the producer (*p* = 0.000), with values ranging from 91.92% to 98.02%. Autumn showed the highest moisture content values (97.08–98.02%), followed by winter (94.65–96.25%) and summer (92.74–94.12%), and the lowest content was found in spring (91.92–92.74%). There are also differences among mezcal producers. La Escondida had a higher moisture content than La Cascada and El Palmar. These results are slightly higher than what was reported by Requena et al. [21], where the bagasse of green and ashen agave was around 80%. This difference stems from bagasse collected after mezcal fermentation and Malinalco’s semi-warm, subhumid climate with summer rainfall.

Factors such as soil pH, the age and species of the agave, the plant’s nutritional status, and the season of the sample collection influence the ash content (*p* = 0.000). The average ash content ranged from 6.34 to 17.75%. The highest amount of ash was recorded in spring (6.52–17.75%), followed by autumn (6.34–14.12%) and winter (8.32–14.73%), with statistically equal amounts, and summer (8.76–9.2%) presented the lowest content. Dias et al. [22] analyzed how the summer and winter seasons affected the nutritional value of *Opuntia ficus-indica* and *Agave americana* L. The study found that summer had a lower ash content of 10.97% compared to winter with 11%. Our findings showed an average ash content higher than that reported by Requena et al. [21], who analyzed six species of desert dietary fiber sources from Mexico. The authors reported a 5% ash content for green agave bagasse and 7% for ashen agave bagasse from the aguamiel. Another study by Escobedo-García et al. [7] found an ash content of less than 10% in agave bagasse from aguamiel.

The lipid content varied from 2.03 to 11.73%. The season with the highest amount of lipids was summer (8.09 to 11.73%), followed by autumn (3.78 to 5.8%) and spring (3.15 to 4.75%), which had similar levels. Winter was the season with the lowest amount of lipids (2.03 to 2.61%). Regarding the producers, La Cascada had the highest lipid content (5.70%), followed by La Escondida (5.33%), and El Palmar (4.89%) had the lowest. The lipid content in our study is slightly lower than the 15% reported by Escobedo-García et al. [7] for ashen agave bagasse from aguamiel. In contrast, our findings are higher than those reported by Delgadillo Ruíz et al. [23], who found 0.274% in *Agave salmiana* bagasse and 0.272% in *Agave weberi* cela bagasse. This difference could be attributed to the agave species, as well as the harvesting season and site. As can be seen, there are significant changes throughout the seasons of the year (*p* = 0.000), which aligns with the findings from Dias et al. [22], where the season with the least amount of lipids was winter.

The protein content varied from 2.27 to 5.15%, and was the highest during the summer and autumn, ranging from 2.80 to 5.15% and 3.40 to 4.45%, respectively. Contrastingly, the lowest protein content was found in the spring and winter, ranging from 2.36 to 4% and 2.26 to 3.81%, respectively. A study on *Agave americana* L. reported a 6.3% protein content in summer and 5.16% in winter, higher than the results of this research [22]. This difference may result from our study focusing on analyzing agave bagasse, whereas the previous study reported the protein content of the entire plant and took into account variations in different agave species. Another study found a protein content of less than 5%, which better matches with this study’s findings, and in this case, both studies focused on agave bagasse despite using different agave species [21].

The carbohydrate content ranged from 74.92% to 86.45%. The winter and spring seasons showed similar behavior, with the highest carbohydrate amounts, 79.42 to 86.45% and 78.51 to 86.13%, respectively. Autumn followed with 78.44 to 83.31%, and summer had the lowest amount at 74.92 to 78.03%. These values are higher than those reported by Escobedo-García et al. [7], where ashen agave bagasse contained about 70% carbohydrates and green agave bagasse contained 65% carbohydrates. This difference is primarily attributed to the agave species and the type of agave bagasse used, with one being for aguamiel and the other for mezcal.

The season- and producer-dependent variations observed in the proximate composition of agave bagasse have important implications for its use as a functional food ingredient. The high carbohydrate content in winter and spring makes these seasons optimal for applications requiring energy-rich or structural components, while the elevated lipid and protein levels in summer suggest greater nutritional or emulsifying potential. Furthermore, differences between producers highlight the role of microclimate, agave maturity, soil, and fermentation practices, which must be considered in raw material sourcing and standardization protocols.

### 3.2. Carbohydrate Content

#### 3.2.1. Sugar Content

**Figure 2:**
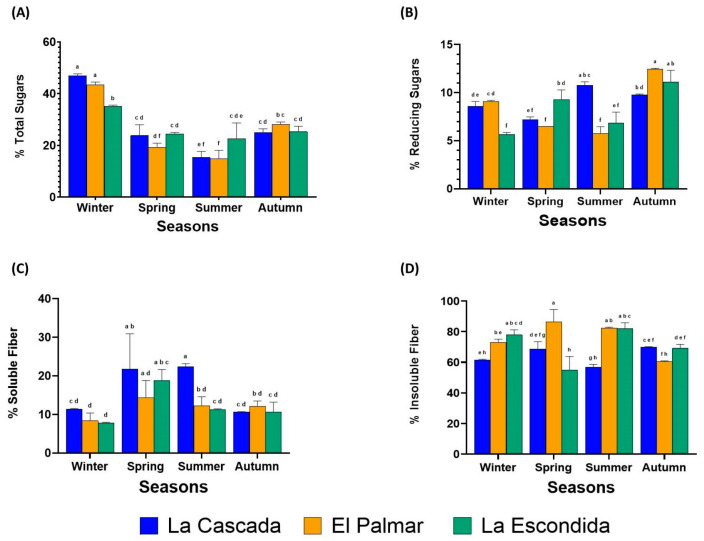
Carbohydrate content in agave bagasse during the four seasons of the year from three different mezcal producers. (**A**) Total sugars (%) using DuBois’ method. (**B**) Reducing sugars (%) using Miller’s method. (**C**) Soluble fiber content (%). (**D**) Insoluble fiber content (%). The data represent the average of three independent experiments ± standard deviation. Distinct letters above the same colored columns indicate significant differences (α = 0.05) based on Tukey’s test, comparing mezcal producers and the four seasons. All results are presented on a dry-weight basis.

Significant variations in total sugar content, ranging from 14.82% to 46.98% (Figure 2A), were observed depending on the season and the mezcal producer. Based on the results of the two-way ANOVA the season had a significant effect on the total sugar content (*p* = 0.000), with winter having the highest amount of sugar (35.08–46.98%), followed by autumn (24.96–28.11%), spring (19.23–24.51%), and summer (14.82–22.68%). The producer did not show significant differences (*p* = 0.450), but the interaction between season and location was significant (*p* = 0.000). La Cascada in winter exhibited the highest total sugar content (46.98%), followed by El Palmar (43.41%) and La Escondida, also in winter (35.08%). These results are higher than those reported by González-García et al. [24] for tequila agave bagasse, and consistent with those of Bhandari et al. [25], who analyzed how the year’s seasons (autumn and spring) affect the sugar concentration in cabbage. Their research showed a lower sugar concentration in autumn and spring, similar to our study. This is mainly because the higher temperatures in spring and summer seasons cause an increase in photorespiration and decrease photosynthesis.

The sugar content is crucial for enhancing the digestibility and usability of lignocellulosic materials for fermentative bacteria. This is particularly important when these materials are used in fermentation processes aimed at upcycling. This approach can facilitate the production of value-added ingredients. Reducing sugars combined with amino acids at high temperatures affects the taste and reduces prebiotic activity [26]. Significant variations were found in reducing sugar content across the seasons and mezcal producers, ranging from 5.66 to 12.46% (Figure 2B). According to the two-way ANOVA, the season significantly and negatively affected the sugar content (*p* = 0.00), with autumn having the highest amount of reducing sugars at 9.80–12.46%, followed by summer, winter, and spring, which showed no significant differences between them. The relation between producers and seasons was also significant (*p* = 0.000). El Palmar had the highest reducing sugar content during autumn, followed by La Escondida in autumn and La Cascada in summer. The reducing sugar content was lower than that reported by González-García et al. [24] for tequila bagasse. Similarly, a study on algae [27] found that the concentration of reducing sugars was higher in the autumn season compared to summer, which aligns with our findings. The increase in concentration is likely influenced by factors such as wind speed, soil salinity, pH, temperature, precipitation, photoperiod, and soil nutrients. These factors contribute to the agave plant and bagasse producing a higher concentration of reducing sugars. Concerning agave, a previous study showed that agave bagasse had about 10% reducing sugars and 46% total sugars in February [28], this is consistent with our findings that climatic conditions are a factor that defines the amount of sugars present.

#### 3.2.2. Dietary Fiber Composition

Dietary fiber is an essential nutrient, offering numerous health benefits, such as aiding in cardiovascular health, controlling the glycemic index, promoting bowel movement, controlling appetite, boosting the immune system, and reducing inflammation, having a prebiotic effect and promoting the generation of short-chain fatty acids [29]. Fiber is composed of soluble and insoluble fiber. Quantifying the type of fiber available in agave bagasse is essential for the proper utilization of this by-product.

Soluble fiber reaches the microbiota and can be fermented by bacteria, acting as a prebiotic [30]. The soluble fiber content of mezcal agave bagasse varied from 7.80% to 22.35% (Figure 2C). According to the two-way ANOVA, the season significantly affected the soluble fiber content (*p* = 0.000). The season with the highest amount of soluble fiber was spring 14.32–21.78%, followed by summer 11.23–22.35% and autumn 10.61–12.12%, and the season with the lowest amount of soluble fiber was winter at 7.80–11.40%. Moreover, location also significantly influenced the soluble fiber content (*p* = 0.002). The bagasse from La Cascada had the highest amount of soluble fiber (16.54%), followed by La Escondida (12.12%) and El Palmar (11.75%), which had similar amounts. These differences can be attributed to the different processing conditions followed by each producer, including cooking temperature, fermentation time, and distillation conditions. The interaction between producers and seasons had a significant effect (*p* = 0.043). In this sense, La Cascada exhibited the highest amount of soluble fiber in the summer (22.35%) and in the spring (21.78%) and La Escondida showed the lowest amount of soluble fiber in winter (18.82%). Our results were higher than those reported for green agave bagasse and ashen agave bagasse [21], with less than 10% of soluble fiber. However, we found similar concentrations to those reported by Bautista-Espinoza et al. [31], where the soluble fiber was 13.88% in agave bagasse from tequila. There are no studies on fiber quantification throughout the year’s four seasons for tequila or for mezcal. Based on the observed results, it is likely that temperature and rainfall are the main factors influencing the more significant amount of soluble fiber in agave bagasse, as they affect agave photosynthesis and the behavior of yeasts used in mezcal fermentation.

Insoluble fiber increases intestinal transit [30]. The insoluble fiber in agave bagasse varies based on the producer and the year’s season within the range of 55.03 to 86.56% (Figure 2D); according to the two-way ANOVA, the producer also affects the amount of insoluble fiber (*p* = 0.000). According to the results, El Palmar had the highest insoluble fiber content, followed by La Escondida, and La Cascada had the lowest amount. The insoluble fiber content was also affected by the year’s season (*p* = 0.013). The summer season was the one with the highest amount of soluble fiber, followed by winter and spring, for which there were no significant differences, and the season with the lowest amount of insoluble fiber was autumn. According to the two-way ANOVA, the interaction of season and producer had an impact on the amount of insoluble fiber (*p* = 0.000); the combination with the highest amount was El Palmar in spring at 86.56%, followed by summer with the same producer at 82.26% and La Escondida with summer 82.96%. The results obtained are more significant than those reported by Requena et al. [21], who found less than 50% insoluble fiber in the agave bagasse of aguamiel. Also, our results are superior to what Bautista-Espinoza et al. [31] reported for agave bagasse from tequila, which contained 65.24% insoluble fiber; this is because the production of tequila is different from that of mezcal, as is the species of agave. Meanwhile, for the season, there is no pattern of high temperatures or precipitation that causes a more significant amount of insoluble fiber, so differences are probably mainly due to the behavior of yeasts.

### 3.3. Bioactive Characterization

#### 3.3.1. Phenol Composition

**Figure 3:**
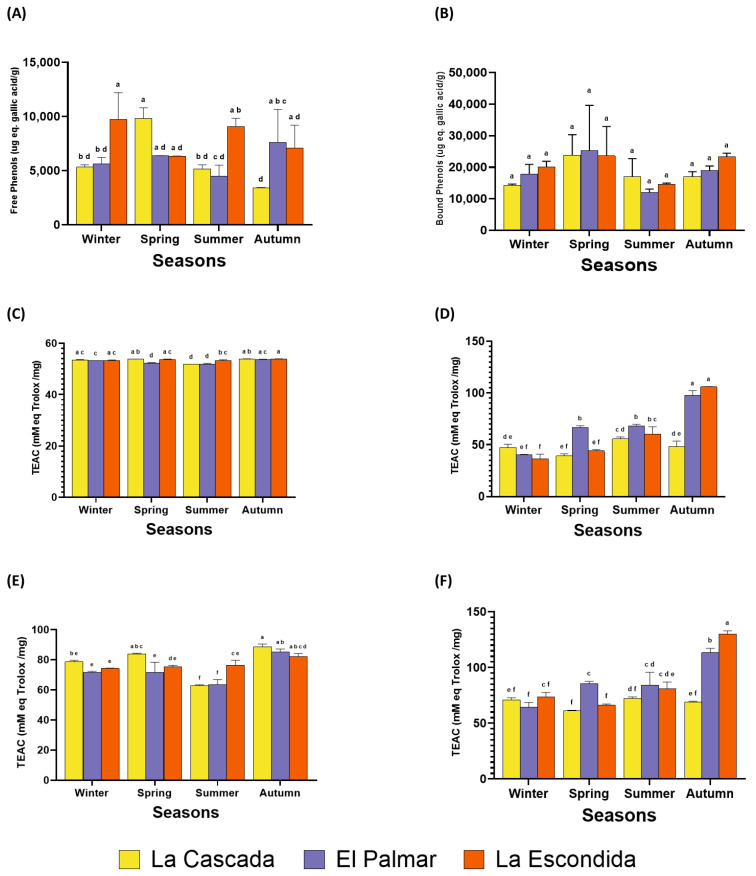
The composition of phenols and antioxidant activity, as assessed by the ABTS and DPPH assays, were analyzed in agave bagasse collected across all four seasons of the year from three mezcal producers. (**A**) Results for free phenols, expressed in µg equivalent of gallic acid per gram, measured using the Folin–Ciocalteu reagent. (**B**) Results for bound phenols, also expressed in µg equivalent of gallic acid per gram, using the same reagent. The antioxidant activity is presented as TEAC (Trolox equivalent antioxidant capacity) in mM of Trolox per milligram of sample. (**C**) DPPH results for the free phenolic fraction. (**D**) DPPH results for the bound phenolic fraction. (**E**) ABTS results for the free phenolic fraction. (**F**) ABTS results for the bound phenolic fraction. Data represent the mean of three independent experiments ± standard deviation. Letters within the same color group of columns indicate significant differences (α = 0.05) based on Tukey’s test between the mezcal producers and seasons. All results are presented on a dry-weight basis.

Phenolic compounds are among the most abundant secondary metabolites in fruits, vegetables, and plants [32]. Phenolics present some health benefits, such as aiding insulin resistance, modulating lipid and carbohydrate metabolism, reducing hyperglycemia, combating oxidative stress, influencing adipose tissue metabolism and inflammatory processes, and serving as prebiotics [33]. These compounds exist in free or bound phenolic form. The main difference is that they are soluble in organic compounds and are absorbed in the small intestine. On the other hand, bound phenols interact with specific macromolecules, are absorbed in the colon, resist enzymatic hydrolysis, and serve as prebiotics [34]. In agave bagasse from the mezcal industry, the free phenol content varied from 3407.97 to 9840.70 μg eq gallic acid/g across different seasons of the year and mezcal producers (Figure 3A). According to the two-way ANOVA, the season did not show significant differences (*p* = 0.126).

In contrast, the producers did not show a significant difference (*p* = 0.001), with La Escondida having the highest amount of free phenols, followed by the other two producers, which did not have significant differences. The interaction between producer and season did have significant differences according to the two-way ANOVA (*p* = 0.000), with La Cascada in spring and La Escondida in winter having the highest content of free phenols at 9840.70 and 9738.92 μg eq gallic acid/g, respectively, followed by La Escondida in summer with 9073.04 μg eq gallic acid/g and El Palmar in autumn with 7614.81 μg eq gallic acid/g. There are no previous studies on quantifying free phenols in agave bagasse. These results are consistent with a study conducted on Opuntia ficus-indica [35], which showed that phenols were present in a higher concentration in spring, followed by winter, summer, and autumn. Our producer and season interactions follow this same pattern. Climate, environment, and soil type influence these variations.

In contrast to the bound phenol content ranging from 11,976.0 to 25,312.8 μg eq gallic acid/g (Figure 3B), the two-way ANOVA did not show significant differences in terms of the season of the year in which the samples were collected (*p* = 0.556). However, the mezcal producer had substantial differences (*p* = 0.010), with spring (24,258.2 μg eq gallic acid/g) having the highest content of bound phenols, followed by the other three seasons of the year that did not have significant differences. The interaction between the producer and the season of the year was not significant (*p* = 0.712). It is also worth noting that spring exhibited the highest concentration of bound phenols, due to the soil conditions favoring their development, and free phenols [36].

While the season alone did not show significant differences, the interaction with the production site was highly significant for free phenols. This indicates that climatic conditions, soil type, and artisanal practices influence phenolic profiles. These findings support the strategic selection of producer–season combinations to maximize bioactive content, which is crucial for designing functional foods or supplements to improve metabolic health.

#### 3.3.2. Antioxidant Capacity

The antioxidant capacity in foods with prebiotic potential is important because antioxidants neutralize free radicals, reducing oxidative stress in the body. They also help with food preservation. A good combination of prebiotics and antioxidants increases the health of the microbiota, which is beneficial for the immune system as it helps reduce inflammation [37].

The two-way ANOVA showed that the season (*p* = 0.000), producer (*p* = 0.000), and their interaction (*p* = 0.000) significantly influenced the content of the antioxidant capacity of free and bound phenols in both the DPPH and ABTS tests. The antioxidant capacity with DPPH was higher for both free and bound phenolic compounds (Figure 3). The values ranged from 62.82 to 88.73 mM eq Trolox/mg for the antioxidant activity of free phenols with DPPH (Figure 3C). The season that had the highest amount of antioxidant activity was autumn (85.32 mM eq Trolox/mg), followed by spring (76.85 mM eq Trolox/mg) and winter (74.76 mM eq Trolox/mg), which had no significant differences, and summer (67.51 mM eq Trolox/mg), which was the lowest season. As for mezcal producers, La Cascada and El Palmar behaved similarly, followed by La Escondida. The antioxidant capacity for the bound fraction (Figure 3D) ranged from 61.3 to 130.13 mM eq Trolox/mg. The season with the highest antioxidant capacity content for the bound fraction was autumn (104.13 mM eq Trolox/mg), followed by summer (79.19 mM eq Trolox/mg), while the lowest amounts were found in spring and winter (70.52 mM eq Trolox/mg), which behaved similarly. The producer with the most minor content was La Cascada; the other two producers behaved similarly.

In determining the antioxidant capacity by ABTS for the free fraction (Figure 3E), the range was from 51.80 to 53.87 mM eq Trolox/mg. Antioxidant activity was highest in autumn (53.78 mM eq Trolox/mg), followed by spring and winter, which had no significant differences (53.30 mM eq Trolox/mg). Summer (52.31 mM eq Trolox/mg) had the lowest activity, consistent with the ABTS test. The producer with the highest concentration was La Escondida, followed by La Cascada and El Palmar. For the bound fraction (Figure 3F), the range was 36.69 to 105.95 mM eq Trolox/mg. All seasons showed significant differences, with autumn having the highest concentration (83.84 mM eq Trolox/mg), followed by summer (61.15 mM eq Trolox/mg) and spring (49.95 mM eq Trolox/mg), with winter (41.51 mM eq Trolox/mg) being the lowest. The producers also showed different behavior, with El Palmar having the highest concentration, followed by La Escondida, and La Cascada having the lowest concentration.

### 3.4. Vibrational Analysis

**Figure 4:**
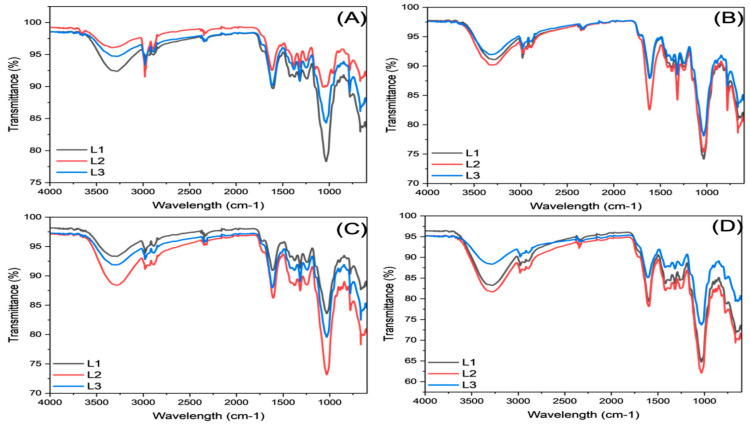
Infrared spectra of agave bagasse from 3 mezcal producers (L1, L2, L3) during the 4 seasons of the year. (**A**) Spring, (**B**) summer, (**C**) autumn, (**D**) winter.

Figure 4 shows the infrared spectrum of agave bagasse samples from different mezcal producers during the four seasons of the year (spring, summer, autumn, and winter). The differences observed in the IR spectra between the mezcal-producing locations and seasons reflect variations in the chemical composition of the agave bagasse, likely due to environmental factors such as humidity, temperature, precipitation, nutrient availability, and bioactive compound accumulation. These compositional changes are primarily related to carbohydrates (e.g., lignin, cellulose), phenolic compounds, and other organic components present in agave bagasse, which fluctuate throughout the year.

Regardless of the season or mezcal-producing location, consistent bands were observed in the 3200–3600 cm^−1^ region, attributed to the stretching vibration of O-H groups. This region is dominated by polysaccharides such as cellulose and hemicellulose, and the differences in transmittance observed in these bands may be associated with varying amounts of hydroxyl groups in these polysaccharides. Notably, more pronounced bands were observed in samples from El Palmar (L2) during the spring and summer, indicating a higher accumulation of structural carbohydrates and phenolics, consistent with the differences in carbohydrate content observed in the proximate composition. This behavior may also be linked to the climatic variations during these seasons, where higher precipitation and temperatures promoted the accumulation of these compounds.

Another region of interest is the 1600–1750 cm^−1^ region, associated with the stretching vibrations of the carbonyl groups (C=O) from reducing sugars, lignin, and lipids. During summer, bands in this region were more pronounced, coinciding with an increase in the content of lipids and free phenolics, particularly at La Escondida (L3). This increase in absorption (lower transmittance) may be linked to high metabolic activity during the summer, favored by warm and humid conditions that promote the synthesis and accumulation of bioactive compounds in agave. Additionally, the aromatic ring vibrations and carboxylic groups of phenolics may also contribute to the absorption in this region. This is particularly relevant as the highest content of free phenolics was observed in the spring and summer.

The 1000–1200 cm^−1^ region is crucial for identifying structural carbohydrates such as cellulose and hemicellulose. In this region, pronounced bands indicating the presence of structural carbohydrates were observed across all seasons and locations. These bands are associated with the vibrations of C-O-C and C-O-H groups in these structural polysaccharides. Samples from La Escondida (L3) collected during autumn showed the most significant absorption (lower transmittance) in this region, likely due to the high concentration of carbohydrates observed in this season (Table 1). The dry and fresh climatic conditions in autumn and winter, along with reduced precipitation, may have favored the accumulation of structural carbohydrates in the agave bagasse.

Overall, noticeable differences were observed in the infrared spectra depending on environmental factors and production practices at the different locations. The spectral changes reflected significant alterations in the chemical composition of agave bagasse, highlighting the influence of the season. A tendency toward greater absorption in key bands associated with bioactive compounds was observed in samples obtained during the spring and summer seasons, while structural carbohydrates were more prominent in colder and drier seasons (autumn–winter). This suggests that spring and summer harvests may be more suitable for applications emphasizing antioxidant and prebiotic properties, while autumn and winter bagasse may be more appropriate for structural or technological functions in food matrices (e.g., texture modification, encapsulation, stabilization).

### 3.5. Principal Component Analysis

**Figure 5:**
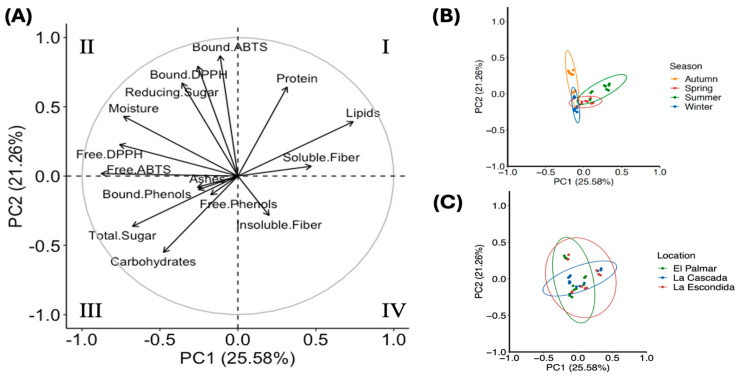
Complete PCA analysis of bioactive components (**A**), PCA analysis by season (**B**) and PCA analysis by producer (**C**).

Figure 5 illustrates the principal component analysis (PCA) performed on the measured data. The first two principal components (PC1 and PC2) account for 46.84% of the total variability in the model, with five principal components required to explain 80% of the variance. No clear separation is observed when the data are grouped by location, as the data from the three producers overlap. However, a clear clustering is observed when the data are grouped by seasonality. The results from summer and autumn are more distant compared to those from spring and winter, potentially due to the closer proximity of the latter seasons. This pattern has been similarly observed in studies on other plants [11], where seasonal variations in environmental factors, such as temperature and rainfall, significantly impact the chemical composition of agricultural products.

The eigenvalue analysis revealed that antioxidant capacity, moisture content, and lipid levels had the greatest influence on PC1, while antioxidant capacity, protein content, and soluble sugars were the most influential on PC2. The loading plot indicates a clustering of moisture, reducing sugars, and antioxidant capacity for the free and bound phenolic fraction, which correlates negatively with insoluble fiber content. Additionally, protein, lipid, and soluble fiber content are negatively correlated with carbohydrate and total sugar content. Samples collected during autumn tend to exhibit higher moisture, reducing sugars, and antioxidant activity from the bound phenolic fraction, whereas samples from summer show higher lipid and soluble fiber content.

Figure 5B shows that compound variation exhibits better clustering, with observable differences among seasons, whereas variability within locations was not enough to yield clear separation of variables. From Figure 5B we observed a clear separation of compounds for autumn (yellow dots, quadrant II), particularly, for reducing sugars and the antioxidant capacity of bound phenolics, that correspond to samples from El Palmar and La Escondida in Figure 5C. Similarly, compounds for summer, corresponding to soluble fiber, were clustered separately in quadrant I for La Cascada and La Escondida.

## 4. Conclusions

The composition of agave bagasse varies significantly by season and location, influenced by climate and production practices. In certain seasons, agave bagasse carbohydrates may exhibit more robust prebiotic properties. Furthermore, agave bagasse’s phenolic content and antioxidant capacity fluctuate throughout the year, indicating potential seasonal advantages from a nutraceutical perspective. Notably, the highest values for moisture, reducing sugars, and antioxidant capacity were observed in autumn; while ash, carbohydrates, and soluble fiber were highest in spring. Summer favored lipid accumulation and winter favored higher total sugars. Parameters such as protein, insoluble fiber, and phenolics showed no consistent seasonal trend due to producer-specific variation. It is important to acknowledge certain limitations of this study: measures such as an analysis with advanced compound-level identification techniques (e.g., LC/MS, HPLC), geographic focus on more than one region, and sampling by more than a single sample are required to improve the results. Although all agave bagasse samples were collected from the same region, the artisanal mezcal production methods varied slightly among producers. These differences include cooking times, fermentation conditions, and general handling practices. Such variability, inherent to traditional production, introduces experimental noise that cannot be fully controlled without altering the authentic practices.

This study represents the first systematic analysis of the seasonal variability of bioactive compounds in agave bagasse for mezcal. Understanding this variability is essential to guide its use as a source of dietary fiber, sugars, and antioxidants in food formulations. This information could be useful for the selection of harvest seasons or processing adjustments to obtain consistent functional properties, supporting its application in prebiotic ingredients or fiber-enriched products. By valorizing this agro-industrial by-product, the study contributes to circular food systems and promotes sustainable innovation in the food industry. Future research should include in vitro or in vivo evaluation of its prebiotic effects, pilot-scale integration into various food matrices, and detailed profiling of phenolic compounds and their bioavailability to fully understand their role in producing sustainable and healthy foods.

## Figures and Tables

**Table 1:** Chemical composition of *Agave* spp. bagasse obtained from the mezcal industry, analyzed across the 4 seasons of the year and 3 mezcal factories.

Component %	La Cascada				El Palmar				La Escondida			
	Spring	Summer	Autumn	Winter	Spring	Summer	Autumn	Winter	Spring	Summer	Autumn	Winter
Moisture	91.92 ± 0.02 ^a^	92.74 ± 0.12 ^bc^	97.51 ± 0.23 ^gh^	95.5 ± 0.28 ^e^	92.54 ± 0.02 ^b^	93.14 ± 0 ^c^	97.08 ± 0.01 ^g^	94.65 ± 0.02 ^d^	94.11 ± 0.01 ^c^	94.12 ± 0.9 ^d^	98.02 ± 0.03 ^h^	96.25 ± 0.02 ^f^
Ash	15.81 ± 0.36 ^ab^	9.32 ± 0 ^cd^	11.37 ± 0.00 ^c^	14.73 ± 0.84 ^b^	6.52 ± 0.08 ^ef^	8.91 ± 0.21 ^cde^	6.34 ± 0.11 ^f^	8.32 ± 0.17 ^def^	17.75 ± 0.3 ^a^	8.76 ± 0.29 ^def^	14.12 ± 0.15 ^b^	8.68 ± 0.76 ^def^
Lipids	4.75 ± 0.1 ^cd^	11.73 ± 0.38 ^a^	4.32 ± 0.30 ^cdef^	2.03 ± 0.12 ^g^	3.15 ± 0.06 ^defg^	8.09 ± 0.58 ^b^	5.8 ± 0.18 ^c^	2.51 ± 0.24 ^fg^	4.67 ± 0.03 ^cde^	10.25 ± 0.11 ^a^	3.78 ± 0.00 ^cdefg^	2.61 ± 0.05 ^efg^
Protein	4.01 ± 0.05 ^c^	3.74 ± 0.02 ^cd^	3.40 ± 0.04 ^d^	3.81 ± 0.06 ^c^	2.73 ± 0.13 ^ef^	5.15 ± 0.01 ^a^	4.45 ± 0.19 ^b^	2.13 ± 0.15 ^ef^	2.36 ± 0.05 ^fg^	2.80 ± 0.05 ^e^	3.64 ± 0.03 ^cd^	2.26 ± 0.06 ^g^
Carbohydrates	78.51 ± 0.71 ^c^	74.92 ± 0.36 ^d^	80.89 ± 0.26 ^bc^	79.42 ± 0.68 ^c^	86.13 ± 0.33 ^a^	77.47 ± 0.57 ^cd^	83.31 ± 0.61 ^ab^	86.44 ± 0.33 ^a^	86 ± 0.71 ^a^	78.03 ± 0.46 ^cd^	78.44 ± 0.14 ^c^	86.46 ± 0.69 ^a^

Data represent the average of three independent experiments ± standard error. All results are expressed on a dry-weight basis. Different letters within the same column indicate significant differences (α = 0.05) according to Tukey’s test, comparing seasons and agave bagasse producers in a two-way ANOVA.

## Data Availability

The original contributions presented in the study are included in the article, further inquiries can be directed to the corresponding author.
